# The Complete Chloroplast Genome of *Arabidopsis thaliana* Isolated in Korea (Brassicaceae): An Investigation of Intraspecific Variations of the Chloroplast Genome of Korean *A. thaliana*

**DOI:** 10.1155/2020/3236461

**Published:** 2020-09-05

**Authors:** Jongsun Park, Hong Xi, Yongsung Kim

**Affiliations:** ^1^InfoBoss Inc., 301 Room, 670, Seolleung-ro, Gangnam-gu, Seoul, Republic of Korea; ^2^InfoBoss Research Center, 301 Room, 670, Seolleung-ro, Gangnam-gu, Seoul, Republic of Korea

## Abstract

*Arabidopsis thaliana* (L.) Heynh. is a model organism of plant molecular biology. More than 1,700 whole genome sequences have been sequenced, but no Korean isolate genomes have been sequenced thus far despite the fact that many *A. thaliana* isolated in Japan and China have been sequenced. To understand the genetic background of Korean natural *A. thaliana* (named as 180404IB4), we presented its complete chloroplast genome, which is 154,464 bp long and has four subregions: 85,164 bp of large single copy (LSC) and 17,781 bp of small single copy (SSC) regions are separated by 26,257 bp of inverted repeat (IRs) regions including 130 genes (85 protein-coding genes, eight rRNAs, and 37 tRNAs). Fifty single nucleotide polymorphisms (SNPs) and 14 insertion and deletions (INDELs) are identified between 180404IB4 and Col0. In addition, 101 SSRs and 42 extendedSSRs were identified on the Korean *A. thaliana* chloroplast genome, indicating a similar number of SSRs on the rest five chloroplast genomes with a preference of sequence variations toward the SSR region. A nucleotide diversity analysis revealed two highly variable regions on *A. thaliana* chloroplast genomes. Phylogenetic trees with three more chloroplast genomes of East Asian natural isolates show that Korean and Chinese natural isolates are clustered together, whereas two Japanese isolates are not clustered, suggesting the need for additional investigations of the chloroplast genomes of East Asian isolates.

## 1. Introduction

*Arabidopsis thaliana* (L.) Heynh. is a well-known species familiar to those who study plant molecular biology as well as genetic engineering. It was considered to be a weedy species before being used as a model organism [1], representing a good example of the usefulness of weeds. Owing to its importance as a model plant organism, its complete chloroplast genome of Col0 strain was deciphered in 1999 [2]. Its length is 154,478 bp, with a large single copy (LSC) region of 84,170 bp and a small single copy (SSC) region of 17,780 bp separated by inverted repeat (IR; 26,624 bp) regions. It was also found to have 128 genes consisting of 87 protein-coding, 37 tRNA, and four rRNA genes.

Consequently, the whole genome sequences of *A. thaliana* were released in 2000, presenting a 115.4 Mb genome with 25,498 genes [3]. Owing to the rapid development of sequencing technologies, including next-generation sequencing (NGS) technologies, more than 1,750 *A. thaliana* whole genome sequences have been sequenced and analyzed; whole genomes of the Bur-0 and Tsu-1 strains were sequenced with an early version of the Illumina sequencer [4]. The genomes of ebi-1 and Ws-2, which are clock mutants, were also sequenced [5]. Whole genomes of 80 strains isolated from eight regions were also sequenced [6]. In addition, whole genomes of 18 *Arabidopsis* ecotypes were sequenced along with providing assembled sequences for each ecotype, which can be used in further comparative genomic analyses [7]. However, the organelle genomes of 1the 8 ecotype genomes were not correctly assembled, though these can be rectified using raw sequences. With additional improvements in NGS technologies that have lowered the costs of sequencing, the number of sequenced *A. thaliana* isolates has been increased over time. Specifically, genomes of 180 *Arabidopsis* lines isolated from Sweden were sequenced [8]. 217 *Arabidopsis* individual genomes to uncover genome-wide methylation patterns were also sequenced [9], and the genomes of 118 Chinese *Arabidopsis* strains were sequenced showing that the Yangtze River population in China can be considered as an independent lineage on the same level of Central Asian and European isolates [10], and 1,135 natural isolates from all over the world were sequenced and analyzed [11]. Interestingly, except for one study on the sequencing Chinese *A. thaliana* strains [10], only two *Arabidopsis* strains originated from East Asia have been sequenced (Kyoto and Tsu0 from Japan) [11]. Therefore, once any genome sequences of *Arabidopsis* isolated in Korea are available, they can serve as a bridge connecting between China and Japan as part of the effort to construct the evolution history of natural isolates of *A. thaliana* in East Asia.

Although more than 1,750 *Arabidopsis* genomes have been sequenced, chloroplast genomes from these strains have not been assembled to ascertain the sequence variations of the chloroplast genome except for Ler-0 [12]. This is likely to be due to the fact that the chloroplast genome does not contain enough information to attract researchers comparing with whole genome sequences. However, chloroplast genomes are occasionally useful for unravelling corresponding phylogenetic relationships based on the maternal lineage: e.g., *Lindera* genus [13], *Fagopyrum* genus [14], *Potentilla* genus [15–21], *Pseudostellaria* genus [22–26], and *Dysphania* genus [27–30]. Moreover, several studies that described assembled organelle genomes from NGS raw sequences generated with different purposes have been conducted [31, 32].

To understand characteristics of *A. thaliana* isolated in Korea (termed 180404IB4) based on chloroplast genome sequences, we completed its chloroplast genome, presenting the third chloroplast genome of *A. thaliana* based on the NCBI database and related publications [2, 12]. The chloroplast genome of *A. thaliana* 180404IB4 presented the shortest total length and the shortest inverted repeat (IR) region length, caused by a 6 bp deletion on *ycf2*. Due to the limitation of available chloroplast genomes of *A. thaliana* (only two are available in NCBI: Col0 and Ler-0), we assembled three additional chloroplast genomes of East Asian isolates of *A. thaliana* from raw sequences downloaded from the Short Read Archive (SRA) in NCBI, indicating that Tsu0 (Japanese isolate) had the longest length due to an approximately 500 bp insertion. Numbers of sequence variations calculated based on the 180404IB4 chloroplast genome are in the middle of the numbers of intraspecific variations of many plant chloroplast genomes. Phylogenetic analyses of these chloroplast genomes indicate that 180404IB4 (Korea) and 11-15 (Chinese) are clustered, whereas two Japanese isolates (Kyoto and Tsu0) are scattered. These results will provide a glimpse of the evolutionary history of *A. thaliana* in the East Asian area region together with upcoming research results.

## 2. Materials and Methods

### 2.1. DNA Extraction of Korean *A. thaliana* Natural Isolate

The Korean *A. thaliana* natural isolate was collected in Yeonggwang-gun, Junranam Province, South Korea (35.242862N, 126.508987E; 180404IB4; Y. Kim, IB-00583, InfoBoss Cyber Herbarium (IN)) in 2018. Its total DNA was extracted from fresh leaves using a DNeasy Plant Mini Kit (QIAGEN, Hilden, Germany).

### 2.2. Genome Sequencing and *De Novo* Assembly of the Korean Natural Isolate *A. thaliana* Chloroplast Genome

Genome sequencing was performed using HiSeqX at Macrogen Inc., Korea, from the extracted DNA of the Korean *A. thaliana de novo* assembly, with confirmation accomplished with Velvet 1.2.10 [33] after filtering raw reads using Trimmomatic 0.33 [34]. After obtaining the first draft of the chloroplast genome sequences, gaps were filled with GapCloser 1.12 [35] and all bases from the assembled sequences were confirmed by checking each base in the alignment (tview mode in SAMtools 1.9 [36]) against the assembled chloroplast genome generated with BWA 0.7.17 [37]. All these bioinformatic processes were conducted under the environment of Genome Information System (GeIS; http://geis.infoboss.co.kr/; Park et al., in preparation).

### 2.3. *De Novo* Assembly of the Japanese and Chinese Natural Isolates *A. thaliana* Chloroplast Genomes

Raw sequences downloaded from NCBI SRA (SRR492307; Kyoto (Japan), ERR031555; Tsu0 (Japan) and SRR2204166; 15-11 (China)) [7, 9, 10] were used for chloroplast *de novo* genome assembly with Velvet 1.2.10 [33] after filtering raw reads using Trimmomatic 0.33 [34] under the environment of Genome Information System (GeIS; http://geis.infoboss.co.kr/; Park et al., in preparation). The remaining steps for finalizing the chloroplast genomes are identical to those used in the assembly process of the Korean *A. thaliana*.

.

### 2.4. Chloroplast Genome Annotation

Geneious R11 11.0.5 (Biomatters Ltd., Auckland, New Zealand) was used for chloroplast genome annotation based on the *A. thaliana* chloroplast genome (NC_000921) [2] by transferring annotations while correcting exceptional cases, including missing start or stop codons. tRNA was predicted and confirmed using tRNAScan-SE [38].

### 2.5. Identification of Sequence Variations on *Arabidopsis* Complete Chloroplast Genomes

Single nucleotide polymorphisms (SNPs) and insertions and deletions (INDELs) were identified from the pairwise alignment of two chloroplast genomes conducted by MAFFT 7.450 [39] in the environment of the Plant Chloroplast Database (PCD; http://www.cp-genome.net/).

### 2.6. Identification of Simple Sequence Repeats (SSRs)

Simple sequence repeats (SSRs) were identified on the chloroplast genome sequence using the pipeline of the SSR database (SSRDB; http://ssr.pe.kr/; Park et al., in preparation). Based on the conventional definition of a SSR on a chloroplast genome, monoSSR (1 bp) to hexaSSR (6 bp), the total length of SSRs on the chloroplast genome exceeds 10 bp. Owing to the different criteria of SSRs on chloroplast genomes [26, 40–45], we adopted the criteria used in chloroplast genome of *Dysphania ambrosioides* [30], as follows: the monoSSR (unit sequence length of 1 bp) to hexaSSR (6 bp) are used as normal SSRs, and heptaSSR (7 bp) to decaSSR (10 bp) are defined as extendedSSRs. Among the normal SSRs, pentaSSRs and hexaSSRs for which the number of unit sequences is 2 are classified as potentialSSRs.

### 2.7. Comparison of SSRs Identified from Six *A. thaliana* Chloroplast Genomes

SSRs identified from six *A. thaliana* chloroplast genomes were compared based on their flanking sequences under the environment of the SSRDB (http://ssr.pe.kr; Park et al., in preparation). The pipeline of the SSR comparison implemented in the SSRDB was used with the following conditions: a cut-off *e* value of 1*e* − 10 and a maximum flanking sequence for the comparison of 60 bp. This comparison was utilized in a comparative analysis of *Chenopodium* chloroplast genomes (Park et al., in preparation) and *Stegobium paniceum* (Park et al., under revision) and *Figulus binodulus* (Lee et al., [46]) mitochondrial genomes.

### 2.8. Nucleotide Diversity Analysis

Nucleotide diversity was calculated using the method proposed by Nei and Li [47] based on the multiple sequence alignment of *Arabidopsis* chloroplast genomes using a Perl script. The window size was set to 500 bp and the step size was 200 bp when using the sliding window method. Genomic coordination of each window was compared to the gene annotation of the chloroplast genome under the environment of PCD environment for further analyses.

### 2.9. Construction of Phylogenetic Trees

Whole chloroplast genomes of seventeen *Arabidopsis* genomes and one *Arabis* chloroplast genome were aligned by MAFFT 7.450 [39], and alignment quality was checked manually. The neighbor-joining (NJ) and maximum likelihood (ML) trees were reconstructed in MEGA X [48]. In the ML analysis, a heuristic search was used with nearest-neighbor interchange (NNI) branch swapping, Tamura-Nei model, and uniform rates among sites. All other options used the default settings. Bootstrap analyses with 1,000 pseudoreplicates were conducted with the same options. The posterior probability of each node was estimated by Bayesian inference (BI) using the Mr. Bayes 3.2.6 [49] plug-in implemented in Geneious R11 11.0.5. The HKY85 model with gamma rates was used as a molecular model. A Markov chain Monte Carlo (MCMC) algorithm was employed for 1,100,000 generations, sampling trees every 200 generations, with four chains running simultaneously. Trees from the first 100,000 generations were discarded as burn-in.

## 3. Results and Discussions

### 3.1. Complete Chloroplast Genome of the First Korean Isolate of *A. thaliana* and Comparison with *A. thaliana* Chloroplast Genomes Assembled from NGS Raw Reads

**Figure 1:**
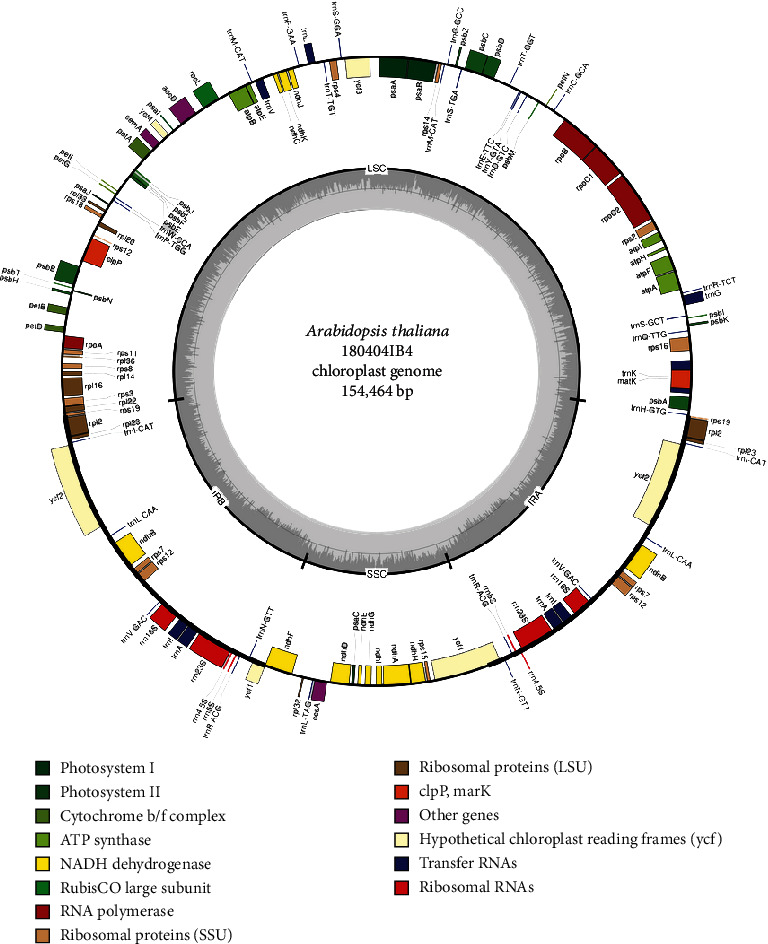
Complete chloroplast genome of Korean isolate of *A. thaliana*, 180404IB4. The genes located outside of the circle are transcribed clockwise, while those located inside are transcribed counter clockwise. The dark grey plot in the inner circle corresponds to GC content. Large single copy, small single copy, and inverted repeat are indicated with LSC, SSC, and IR (IRA and IRB), respectively.

The chloroplast genome of *A. thaliana* 180404IB4 (GenBank accession number is MK353213) is 154,464 bp and has four subregions with 85,164 bp of large single copy (LSC) and 17,781 bp of small single copy (SSC) regions separated by 26,257 bp of inverted repeat (IR; Figure 1). It contains 130 genes (85 protein-coding genes, eight rRNAs, and 37 tRNAs), with 19 genes (8 protein-coding genes, 4 rRNAs, and 7 tRNAs) that are duplicated in IR regions (Figure 1). The overall GC content is 36.3%, and those contents in the LSC, SSC, and IR regions are 34.0%, 29.3%, and 42.3%, respectively.

To determine the characteristics of *A. thaliana* chloroplast genomes from East Asia, we also completed three chloroplast genomes of *A. thaliana*, two from Japan (Tokyo and Tsu0) and one from China (11-15; Table 1). In addition, two available chloroplast genomes (Col0 and Ler-0) were used for comparative analyses. Their lengths range from 154,464 bp (180404IB4) to 154,938 bp (Tsu0) and the IR length ranges from 26,257 bp to 26,264 bp (Table 1). The chloroplast genome of the Korean isolate, 180404IB4, is the shortest, and its IR is also the shortest among four East Asian *A. thaliana* chloroplasts (Table 1). It is caused by 6 bp deletion on *ycf2* located in the IR region compared to the rest five *A. thaliana* chloroplast genomes. Interestingly, Tsu0 shows the longest length of chloroplast genome, caused by an insertion of approximately 500 bp between *trnL* and *trnF*, which is similar to the cases of *Coffea arabica* with one continuous insertion region [50], *Duchesnea chrysantha* showing three continuous insertion regions [21], *Viburnum amplificatum* showing two continuous insertion regions [32], and mitochondrial genomes of *Populus tremula* x *Populus glandulosa* and *Liriodendron tulipifera* with four and thirty-three continuous insertion regions, respectively [51, 52].

The GC content of the six complete chloroplast genomes of the *Arabidopsis* isolates is 36.3% and those of LSC, SSC, and IR are 34.0%, 29.3%, and 42.0%, respectively. An exception is GC content of the LSC of Tsu0, which is 34.1% (Table 1). This is also caused by the inserted region of the Tsu0 chloroplast genome. Other plant chloroplasts of which intraspecific variations of the GC contents are identical to those of *A. thaliana* are *Goodyera schlechtendaliana* (37.1% and 37.2%) [53–55] and *Gastrodia elata* (26.7% and 26.8%) [56–58] which are same to those of *Arabidopsis thaliana*, while *Coffea arabica* [50, 59–63], *Viburnum erosum* [64, 65], *Duchesnea chrysantha* [20, 21], *Salix koriyanagi* [66, 67], *Pseudostellaria palibiniana* [23, 25], and *Pyrus ussuriensis* [68, 69] present no difference in the intraspecific GC contents.

### 3.2. Identification and Evaluation of Sequence Variations of the *A. thaliana* 180404IB4 Chloroplast Genome against the Col0 Chloroplast Genome

Based on the pairwise alignment with the *A. thaliana* Col0 chloroplast genome (GenBank accession is NC_000932), 50 single nucleotide polymorphisms (SNPs) and 14 insertion and deletions (INDELs) are identified. Two SNPs on *rpoC2* and one SNP each on the *ycf2* and *ndhF* genes are nonsynonymous SNPs, while for *rpoC2*, *rpoB*, *rbcL*, *rpl20*, and *psbB*, one SNP in each case is synonymous (Table 2). Specifically, the *ycf2* has a 6 bp deletion on the 180404IB4 chloroplast; this does not cause frameshift but is a critical variation making the 180404IB4 chloroplast shortest among the six chloroplast genomes (Tables 1 and 2). Except for this deletion, all INDELs exist in the intergenic space. These INDELs cause the 180404IB4 chloroplast genome to be shorter than the chloroplast genome of Col0 by 14 bp.

To evaluate the degree of these sequence variations including SNPs and INDELs, we investigated studies of intraspecific variations on chloroplast genomes that checked numbers of SNPs and INDELs (Table 3). Some of these studies are compared chloroplast genomes of natural isolates (e.g., *Duchesnea chrysantha* [21]) and some compared among cultivars to find useful molecular markers (e.g., *Chenopodium quinoa* [70]; Table 3). These studies cover 23 families showing relatively large coverage, so that we expected that some characteristics of these sequence variations on chloroplast genomes can be rescued. In addition, we used number of SNPs and INDELs directly during comparison of sequence variations for better understanding intuitively because their complete chloroplast genome lengths are around 150 kb except genera *Marchantia*, *Selaginella*, *Gastrodia, Illicium*, *Pseudostellaria*, and *Daphne* (Table 3).

The numbers of SNPs and INDELs, 50 and 14, respectively, between 180404IB4 and Col0 are smaller than those of *Marchantia polymorpha* between Korea and Poland (69 SNPs and 660 INDELs) [71], *Camellia japonica* (78 SNPs and 643 INDELs) [72], *Rehmannia glutinosa* (147 SNPs and 87 INDELs) [73], and *Selaginella tamariscina* (1,213 SNPs and 1,641 INDELs) [74] between Chinese and Korean isolates, *Pseudostellaria palibiniana* (84 SNPs and 125 INDELs) [25] and *Pyrus ussuriensis* inside Korea (121 SNPs and 781 INDELs) [68], *Eucommia ulmoides* inside China (75 SNPs and 80 INDELs) [75], some cases of *Cucumis melo* [76] and *Chenopodium quinoa* [70], all of *Dioscorea polystachya* [77], *Oryza sativa* among cultivars [78], *G. schlechtendaliana* [53], and *G. elata* [56] (Table 2). Some of species, such as *Potentilla* (49 SNPs and 17 INDELs are identified from *Potentilla stolonifera* var. *quelpaertensis* and *Potentilla stolonifera* var. *chejuensis*) [16, 18], present numbers of sequence variations similar to that of *A. thaliana* between 180404IB4 and Col0. Considering other cases involving the number of intraspecific variations on the chloroplast genome, including *Salix* (40 SNPs and 139 INDELs between *Salix koriyangai* and *Salix gracilistyla*) [66, 79], *Ilex* (55 SNPs and 429 INDELs between *Ilex cornuta* and *Ilex integra*) [15, 80], and *Nymphaea* (586 SNPs and 1,150 INDELs between *Nymphaea capensis* and *Nymphaea ampla*) [21], no clear levels pertaining to the number of intraspecific or interspecific variations exist. However, the numbers of SNPs and INDELs between 180404IB4 and Col0 are relatively small considering the intercontinental distance between two samples of the same plant species.

### 3.3. Comparison and Evaluation of Sequence Variations of Chloroplast Genomes of the Six *A. thaliana* in East Asia

**Figure 2:**
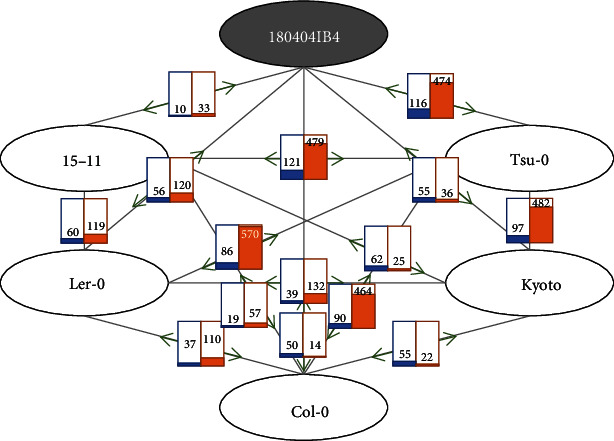
Sequence variation map of six *Arabidopsis thaliana* chloroplast genomes. Eclipses with black border indicate six isolates of *A. thaliana* and blue and orange bar graphs on grey lines connected between two eclipses indicate the numbers of SNPs and INDELs between two isolates. Green thick arrows show two isolates related to the numbers of SNPs and INDELs.

Based on 15 pairwise alignments of the six *A. thaliana* chloroplast genomes, the numbers of SNPs and INDELs between two *A. thaliana* chloroplast genomes range from 10 to 116 and from 22 to 570, respectively (Figure 2). The Tsu0, Japanese natural isolate, chloroplast genome contains large insertions compared to the remaining five chloroplast genomes of *A. thaliana*, supported by the largest Tsu0 chloroplast genome (Table 1). The number of INDELs compared to the Tsu0 chloroplast genome (GenBank accession number is MK380721) ranges from 470 to 570, much higher than those of other combinations (Figure 2). This case is similar to those of *C. arabica*, showing one 84 bp insertion region [50] and *D. chrysantha*, presenting three insertion regions [21]. In terms of the number of INDELs, it is also in relation to high intraspecific variations that only *P. ussuriensis* [68], *G. schlechtendaliana* [53], and *G. elata* [56] present higher numbers of INDELs (Table 3). In addition, two out of the three Orchidaceae species shows high rates of divergence in terms of flower morphologies as well as the number of species [81–83]. This indicates that the Tsu0 insertion is an exceptional case of intraspecific variation. Consequently, Kyoto (GenBank accession number is MK380720), which was also isolated in Japan, and Tsu0 correspondingly present 97 SNPs and 482 INDELs (Figure 2), suggesting that Tsu0 has a different genomic configuration compared to the remaining five strains.

**Figure 3:**
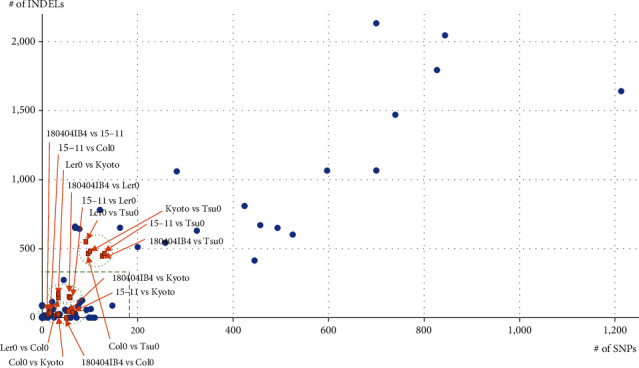
Plot of the numbers of SNPs and INDELs identified as intraspecific variations from chloroplast genomes. *X*-axis indicates the number of SNPs and *Y*-axis means the number of INDELs. Yellow-colored boxes indicate intraspecific comparisons of *A. thaliana* chloroplast genomes. Green dotted circles show a group of intraspecific comparisons. Thick green dotted line displays border between low level of intraspecific sequence variations and high level of intraspecific variations.

The numbers of sequence variations on six *Arabidopsis* chloroplast genomes were plotted together with the numbers of intraspecific variations identified from 90 comparisons of 31 species (Table 3), resulting in three groups; one shows that the number of SNPs is less than 80 and that the number of INDELs is less than 100, the second indicates that the number of SNPs is less than 80 and number of INDELs is between 100 and 200, and the third shows that the number of SNPs exceeds 80 and that the number of INDELs is approximately 500 (Figure 3). The third group is caused by the long insertion of the Tsu0 chloroplast genome. The third group is positioned with a relatively high number of variations areas, while the remaining groups are similar to the most of intraspecific variations on chloroplast genomes (green thick dotted circles in Figure 3).

### 3.4. Comparative Analysis of Simple Sequence Repeats (SSRs) Polymorphisms on Chloroplast Genomes inside East Asian *A. thaliana*

One hundred and one simple sequence repeats (SSRs) and 42 extendedSSRs on the chloroplast genome sequences of the Korean isolate of *A. thaliana* were identified (Supplementary Table 1). One hundred and four (72.72%), 18 (12.59%), and 21 (14.69%) SSRs and extendedSSRs were found in the LSC, IR, and SSC regions, respectively. This distribution is similar to that of *Dysphania ambrosioides*, but not to those of *Dysphania pumilio* or *Dysphania botrys* [30]. Eighteen SSRs and four extendedSSRs (15.38%) are located in the exonic regions of ten protein-coding genes, *mat*K, *trn*K, *trn*R, *rpo*C2, *rpo*B, *atp*B, *acc*D, *psb*B, *rps*12, *rpo*A, *ycf*1, and *ndh*F, and two tRNA genes, which is higher proportion than that of *D. ambrosioides* [30]. In addition, the number of genes on the *A. thaliana* chloroplast genome exceeds that of *D. ambrosioides* by one, while five out of ten protein-coding genes are shared between two species. 25 SSRs and 13 extendedSSRs (26.57%) are in intronic regions of five protein-coding genes and three tRNAs: *ycf*3, *rps*12, *clp*P, *rps*16, and *ndh*A and *trn*K, *trn*R, and *trn*A, respectively. Compared to previous findings that identified SSRs in 12 chloroplast genomes of Brassicaceae, the numbers of SSRs found on the genes are similar to each other, ranging from 40 to 60 [45], which is similar to that of the *A. thaliana* Korean isolate.

We also applied the same method to identify SSRs of the other five chloroplast genomes of *A. thaliana* used in this study (Table 4). The total numbers of SSRs and extendedSSRs range from 143 to 145, showing that the Korean isolate of *A. thaliana* has the fewest, at 143 (Table 4). Based on the number of sequence variations among the six chloroplast genomes (Figure 2), the numbers of SSRs and extendedSSRs along with the motif length are expected to be nearly identical; however, only the triSSRs, nonaSSRs, and decaSSRs show identical numbers across the six chloroplast genomes (Table 4).

**Figure 4:**
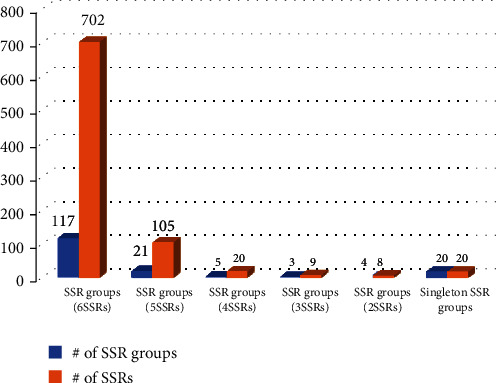
Distribution of the SSR groups identified from six chloroplast genomes of *A. thaliana*. *X*-axis indicates the types of the SSR groups and *Y*-axis means the number of the SSR groups or SSRs/extendedSSRs. Blue graph means the number of the SSR groups and orange bars mean the # of SSRs from the SSR groups.

Using the SSR comparison pipeline implemented in SSRDB, 117 groups of SSRs or extendedSSRs containing six SSRs from the six *A. thaliana* chloroplast genomes are identified, accounting for 702 out of 864 SSRs or extendedSSRs (81.25%; Figure 4). There is one interesting SSR group (named as SSR Group 2) containing six SSRs from the six *A. thaliana* chloroplasts: two are octaSSRs (TATCTATA∗2) and four are diSSRs (TA∗5). Twenty-one SSR groups contain five SSRs or extendedSSRs from five chloroplast genomes, explaining 105 out of 864 SSRs or extendedSSRs (12.15%; Figure 4). Five SSR groups containing four SSRs or extendedSSRs from four chloroplast genomes and three SSR groups covering three SSRs or extendedSSRs from three chloroplasts, four SSR groups having two SSRs or extendedSSRs from two chloroplast, and 20 singletons, indicating unique SSRs, among the six chloroplast genomes are identified (Figure 4). Considering the coverage of the SSRs and extendedSSRs on Korean isolate of the *A. thaliana* chloroplast genome (in total, 1,825 bp out of 154,464 bp; 1.18%), the expected number of sequence variations of the SSR and extendedSSR regions is 0.75; however, the number of common SSRs or extendedSSRs is 117 (81.82%), indicating that the numbers of sequence variations located in SSR or extendedSSR regions are lower than expected number (107.25 sequence variations for SSR or extendedSSR regions). These variations can be used to develop molecular markers [41].

### 3.5. Comparison of Nucleotide Diversity among the Six *A. thaliana* Chloroplast Genomes

**Figure 5:**
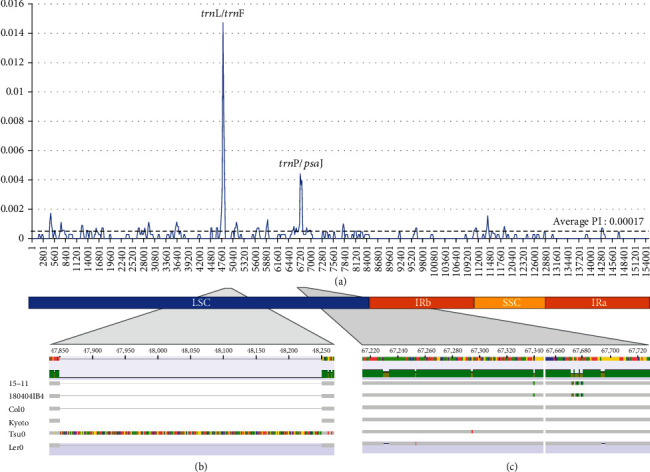
Nucleotide diversity analysis of *Arabidopsis thaliana* chloroplast genomes. (a) *X*-axis means the coordination of alignment of *A. thaliana* chloroplast genomes and *Y*-axis indicates the nucleotide diversity (pi value). Black dotted line means the average value of pi. Three colored boxes show regions of chloroplast genome: LSC, SSC, and IR regions. (b) Enlarged diagram of alignment between 47,830 and 48,280. (c) Two regions between 67,200 and 67,360 and between 67,640 and 67,740.

Nucleotide diversity among six *Arabidopsis thaliana* chloroplast genomes was calculated, indicating that the average nucleotide diversity is 0.00017 (Figure 5(a)), which is at least ten times lower than those of *Dysphania* (0.0068; Chenopodiaceae) [27] and *Viburnum* (0.00176; Adoxaceae) [32]. This is a justifiable result because sequence diversity within species is usually lower than interspecific nucleotide diversity.

There are two significant peaks identified in the sliding window analysis of nucleotide diversity: one is *trn*L/*trn*F (pi value is 0.0147) and the other is *trn*P/*psa*J (pi value is 0.00441). There are fewer peaks than in other studies, including those focusing on *Dysphania* [27] and *Viburnum* [32], stemming from the low level of nucleotide diversity throughout the chloroplast genome. The first peak, *trn*L/*trn*F, appeared due to one large insertion of the Tsu0 chloroplast genome (Figure 5(b)). The second peak, *trn*P/*psa*I, reflects the sequence variations occurring in 180404IB4 (Korea), 15-11 (China), Tsu0 (Japan), and Ler0 (Germany; Figure 5(c)). Specifically, SNPs located between 67,670 and 67,680 in both 180404IB4 and the 15-10 isolates mainly contribute to this peak (Figure 5(c)).

### 3.6. Comparison of the IR Junction among *Arabidopsis thaliana* Chloroplast Genomes

**Figure 6:**
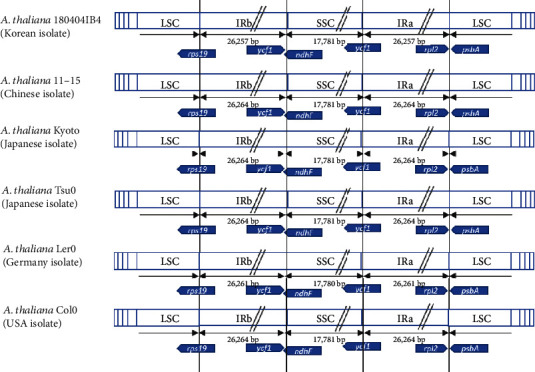
Structure of IR junction of six *Arabidopsis thaliana* chloroplast genomes. Blue diagrams present three chloroplast genomes of *A. thaliana* with each region. Black arrows show length of each region except LSC, and blue arrow diagrams show genes located in junctions between LSC and IRb, IRb and SSC, SSC and IRa, and IRa and LSC.

The IR region on the plant chloroplast genome is the major origin at which to expand or to shrink the chloroplast genome sequences [84–88]. An investigation of the IR junctions of *A. thaliana* chloroplast genomes shows that there are no differences among six *A. thaliana* chloroplast genomes, in agreement with the finding of no structural variations (Figure 6), identical to the case of *D. ambrosioides* [30]. In addition, all *Arabidopsis* chloroplast genomes used in this study present the same structure in the IR junctions.

### 3.7. Phylogenetic Analysis of Korean *A. thaliana* Chloroplast Genome Sequence

**Figure 7:**
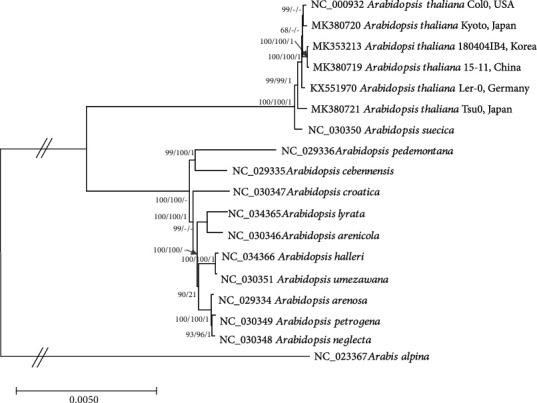
Phylogenetic trees of 18 complete chloroplast genomes of *Arabidopsis* and *Arabis* neighbor-joining (bootstrap repeat is 10,000) and maximum likelihood (bootstrap repeat is 1,000) phylogenetic trees as well as Bayesian inference tree (1,100,000 generations) of fifteen *Arabidopsis* and one *Arabis* from Brassicaceae complete chloroplast genomes: six *Arabidopsis thaliana* (MK353213; 180404IB4, in this study, NC_000932; Col0, KX551970; Ler0, MK380721; Tsu0, MK380720; Kyoto, and MK380719; 15-11), *Arabidopsis suecica* (NC_030350), *Arabidopsis pedemontana* (NC_029336), *Arabidopsis cebennensis* (NC_029335), *Arabidopsis croatica* (NC_030347), *Arabidopsis arenicola* (NC_030346), *Arabidopsis lyrata* (NC_034365), *Arabidopsis umezawana* (NC_030351), *Arabidopsis helleri* (NC_034366), *Arabidopsis arenosa* (NC_029334), *Arabidopsis petrogena* (NC_030349), *Arabidopsis neglecta* (NC_ 030348), and *Arabis alpine* (NC_023367). Phylogenetic tree was displayed based on neighbor-joining tree. The numbers above branches indicate bootstrap support values of neighbor-joining and maximum likelihood phylogenetic trees and posterior possibility value of Bayesian inference tree, respectively.

Bootstrapped neighbor-joining (NJ), maximum parsimony (ML), and Bayesian inference (BI) phylogenetic trees of seventeen *Arabidopsis* chloroplast genomes including six *A. thaliana* chloroplasts and one *Arabis* chloroplast genome as outgroup species indicate that 180404IB4 is clustered with the 15-11 (Chinese natural isolate) with high bootstrap support, while two Japanese isolates are not clustered together in contrast to what was expected here (Figure 7), as Tsu0 has an approximate 500 bp insertion compared to all other *A. thaliana* chloroplast genomes. This indicates that more chloroplast genomes of East Asian *A. thaliana* natural isolates should be investigated to find exceptional sequence variations, such as a Tsu0 insertion. Practically, it is possible to utilize currently available NGS raw read datasets of *A. thaliana* natural isolates by adding an effort to assemble them. In addition, we must consider the possibility of leaking Col0 strains from many molecular laboratories in Korea, which will affect their genetic diversity in some ways. Based on the phylogenetic trees, there appears to be no contamination in Korea.

Several intraspecific phylogenetic relations of plant species using whole chloroplast genomes have been studied, including *Aconitum coreanum*, showing small branches of three individuals with high bootstrap values from both ML and NJ methods from mid-level of sequence variations [89]; *G. schlechtendaliana*, displaying branches of each samples caused by a sufficient number of sequence variations with high bootstraps in both methods [53, 54]; *Abeliophyllum distichum*, indicating partial support of intraspecific individuals from both methods [90–92]; and *Coffea arabica*, showing high bootstrap values from both methods with no branch of either individual sequences due to the low level of sequence variations [50, 59–63]. All these results differ from that of *A. thaliana*, presenting a different clade structure from the phylogenetic trees constructed by three methods (Figure 7). Instead, this phenomenon was found in genome studies focusing on intraspecific variations of insect, fungal, and marine invertebrate mitochondria. These include *Laodelphax striatellus* [93, 94] and *Nilaparvata lugens* [95–97] belonging to the Delphacidae family; *Fusarium oxysporum* which is a fungal plant pathogen [98, 99] and *Apostichopus japonicus* [100]. Because *A. thaliana* has a sufficient amount of sequencing data to construct chloroplast genomes, additional studies with more complete chloroplast genomes will provide a clear answer as to whether or not this phenomenon remains.

In addition, *A. thaliana* and *Arabidopsis suecica* are clustered because the maternal origin of *A. suecica* is *A. thaliana* [101] (Figure 7). Moreover, the topology of BI tree for the remaining *Arabidopsis* species, except for *A. thaliana* and *A. suecica*, is somewhat inconsistent to those of NJ and ML trees (Figure 7), which has also been found in various plant chloroplast genomes [15, 20, 28, 51, 67, 69, 72, 79, 91, 92, 102–109]. This suggests that detailed investigations of phylogenetic relationships among *Arabidopsis* species with various methods should be done in near future.

## 4. Conclusions

We sequenced and assembled the chloroplast genome of the Korean isolate of *A. thaliana* and compared this with the other East Asian *A. thaliana* chloroplast genomes assembled from NGS raw reads available to the public. Based on the numbers of sequence variations of the six *A. thaliana* chloroplast genomes, three groups with low, medium, and high levels of sequence variations were found, particularly due to the large insertion identified on the Tsu0 chloroplast genome. Here, 101 SSRs and 42 extendedSSRs were identified on the Korean *A. thaliana* chloroplast genome, with similar numbers of SSRs on the remaining five chloroplast genomes with a preference of sequence variations of the SSR region. Nucleotide diversity on the six *A. thaliana* chloroplast genomes indicates only two regions that are highly variable, an outcome that is less dynamic than those of interspecific comparisons of chloroplast genomes. As expected, the IR borders of the six chloroplast genomes are conserved. Phylogenetic analyses of the six *A. thaliana* chloroplast genomes with those of other *Arabidopsis* species revealed that the geographical distribution is not congruent with the phylogenetic relationships; however, more complete chloroplast genomes are required for further analysis. Additional whole chloroplast genomes of *A. thaliana* strains based on a large amount of genomic resources of *A. thaliana* can describe the detailed evolutionary history of the natural isolates of *A. thaliana* in East Asia, especially for Korea, China, and Japan.

## Figures and Tables

**Table 1:** List of chloroplast genomes of East Asian *Arabidopsis* strains.

Strain name	Country	GenBank accession	Length (bp)				GC contents			
			Whole	LSC	SSC	IR	Whole	LSC	SSC	IR
180404IB4	Korea	MK353213	154,464	84,170	17,781	26,257	36.3%	34.0%	29.3%	42.3%
Kyoto	Japan	MK380720	154,470	84,162	17,781	26,264	36.3%	34.0%	29.3%	42.3%
Tsu0	Japan	MK380721	154,938	84,630	17,781	26,264	36.3%	34.1%	29.3%	42.3%
11-15	China	MK380719	154,487	84,179	17,781	26,264	36.3%	34.0%	29.3%	42.3%
Col0	USA	NC_000932	154,478	84,170	17,781	26,264	36.3%	34.0%	29.3%	42.3%
Ler-0	Germany	KX551970	154,515	84,213	17,780	26,261	36.3%	34.0%	29.3%	42.3%

**Table 2:** List of genes containing SNPs and INDELs in 180404IB4 strain in *A. thaliana.*

No	Type	Genomic coordination	Gene name	Base changes	Amino acid changes
1	SNP	4,370	Intergenic space	T to A	-
2	SNP	5,705	Intron of *rps*16	C to A	-
3	SNP	5,943	Intron of *rps*16	G to A	-
4	SNP	8,502	Intergenic	T to A	-
5	SNP	12,673	Exon of *atp*F	G to A	Synonymous SNPs
6	SNP	14,825	Intergenic	T to A	
7	SNP	16,380	Exon of *rpo*C2	G to C	C to S
8	SNP	17,801	Exon of *rpo*C2	T to C	Synonymous SNP
9	SNP	17,823	Exon of *rpo*C2	T to G	K to T
10	SNP	23,915	Exon of *rpo*B	T to G	Synonymous SNP
11	SNP	26,817	Exon of *rpo*B	G to C	Synonymous SNP
12	SNP	26,999	Intergenic	T to G	
13	SNP	27,538	Intergenic	C to A	
14	SNP	28,360	Intergenic	T to G	
15	SNP	31,912	Intergenic	A to C	
16	SNP	35,167	Intergenic	G to A	
17	SNP	36,798	Intergenic	T to C	
18	SNP	45,107	Intergenic	G to A	
19	SNP	47,760	Intergenic	T to G	
20	SNP	49,938	Intergenic	T to A	
21	SNP	50,606	Intergenic	T to A	
22	SNP	50,688	Intergenic	T to A	
23	SNP	56,373	Exon of *rbc*L	C to T	Synonymous SNP
24	SNP	58,860	Intergenic	G to C	
25	SNP	65,250	Intergenic	T to C	
26	SNP	65,545	Intergenic	T to G	
27	SNP	66,114	Intergenic	C to A	
28	SNP	66,810	Intergenic	T to C	
29	SNP	67,142	Intergenic	T to A	
30	SNP	67,143	Intergenic	G to A	
31	SNP	67,145	Intergenic	T to A	
32	SNP	67,146	Intergenic	G to C	
33	SNP	67,147	Intergenic	T to A	
34	SNP	67,149	Intergenic	T to C	
35	SNP	67,150	Intergenic	T to A	
36	SNP	68,722	Exon of *rpl*20	T to C	Synonymous SNP
37	SNP	69,347	Intergenic	G to A	
38	SNP	73,507	Exon of *psb*B	T to G	Synonymous SNP
39	SNP	75,386	Intergenic	G to A	
40	SNP	77,830	Intergenic	G to A	
41	SNP	80,688	Intergenic	T to G	
42	SNP	91,824	Exon of *ycf*2	G to C	R to T
43	SNP	96,023	Intron of *ndh*B	T to G	
44	SNP	111,048	Exon of ndhF	T to C	G to R
45	SNP	113,690	Intergenic	T to G	
46	SNP	113,770	Intergenic	T to G	
47	SNP	113,957	Intergenic	C to A	
48	SNP	115,487	Intergenic	T to G	
49	SNP	142,626	Intron of *ndh*B	T to G	
50	SNP	146,825	Exon of ndhF	T to C	G to R
51	INDEL	92,548	Exon of *ycf*2	T to -	DN to -
52	INDEL	92,549	Exon of *ycf*2	G to -	
53	INDEL	92,550	Exon of *ycf*2	A to -	
54	INDEL	92,551	Exon of *ycf*2	T to -	
55	INDEL	92,552	Exon of *ycf*2	A to -	
56	INDEL	92,553	Exon of *ycf*2	A to -	
57	INDEL	108,115	Intergenic	A to -	
58	INDEL	130,525	Intergenic	T to -	
59	INDEL	146,092	Exon of *ycf*2	A to -	DN to -
60	INDEL	146,093	Exon of *ycf*2	T to -	
61	INDEL	146,094	Exon of *ycf*2	C to -	
62	INDEL	146,095	Exon of *ycf*2	A to -	
63	INDEL	146,096	Exon of *ycf*2	T to -	
64	INDEL	146,097	Exon of *ycf*2	T to -	

**Table 3:** List of intraspecific variations of plant chloroplast genomes.

Family	Species	Total length (bp)	# of SNPs	# of INDELs	Comparison samples		Ref.
Marchantiaceae	*Marchantia polymorpha*	120,288~120,304	4	0	Samples isolated in Korea	Kitashirakawa-2 (Japan)	[71]
			0	0	Takaragaike-1 (Japan)	Kitashirakawa-2 (Japan)	
			69	660	MG762001 (Poland)	Kitashirakawa-2 (Japan)	
Selaginellaceae	*Selaginella tamariscina*	126,368~126,700	1,213	1,641	Sample isolate in Korea	Sample isolate in China	[74]
	*Selaginella uncinate*	144,161~144,710	525	602	Sample isolate in Japan	Sample isolate in China	
Magnoliaceae	*Magnolia kobus*	159,411~159,443	50	50	Sample isolate in Korea (Jeju Island)	Sample isolate in Korea (Jeju Island)	In prep.
	*Liriodendron tulifipera*	159,886	12	0	Sample isolate in Korea	Sample isolate in China	[52]
Nymphaeaceae	*Nymphaea alba*	159,25~159,930	11	6	Sample imported in Korea	Sample isolated in Germany	[16]
Schisandraceae	*Illicium anisatum*	142,723~142,747	21	114	Sample isolated in Korea	Sample isolated in USA	[102]
Rubiaceae	*Coffea arabica*	155,186~155,277	2	2	Cold hardness coffee tree (CH3) planted in Jeju Island	NC_008535	[62]
			0	2	Coffee tree showing high productivity (HP1) in Jeju Island	NC_008535	[61]
			0	84	Coffee tree imported to Korea from Indonesia in near to 30 years ago (IN1)	NC_008535	[50]
			3	8	NC_008535	*Coffea arabica* “Typica”	[59]
			0	3	KY085909		
			0	4	MK342634, CH3 isolate		
			0	4	HP1 isolate		
			0	90	IN1 isolate		
			3	6	NC_008535	*Coffea arabica* “Typica Bluemoutain”	[60]
			0	1	KY085909		
			0	6	MK342634, CH3 isolate		
			0	6	HP1 isolate		
			0	88	IN1 isolate		
			0	2	*Coffea arabica* “Typica”		
			0	0	CH1 isolate	CH2 isolate	[63]
Theaceae	*Camellia japonica*	156,606~157,047	25	2	Sample isolated in Soyeonpyeong-do, Incheon, Korea	Wimi-ri in Jeju, Korea	[72]
			78	643	Sample isolated in Soyeonpyeong-do, Incheon, Korea	Sample isolated in China	
			1	1	Sample isolated in Seogwang-ri, Jeju, Korea	Sample isolated in Soyeonpyeong-do, Incheon, Korea	[105]
Caryophyllaceae	*Pseudostellaria longipedicellata*	149,668	0	0	Sample isolate in Mt. Taebak, Korea	Sample isolate in Mt. Taebak, Korea	Unpub.
	*Pseudostellaria palibiniana*	149,639~149,668	84	125	Sample isolated in Mt. Taebaek, Korea	Sample isolated in Mt. Gwangdeok, Korea	[25]
Salicaceae	*Salix koriyanagi*	155,548	0	0	Male sample in Seoul, Korea	Female sample in Seoul, Korea	[67]
Rosaceae	*Agrimonia pilosa* ^∗^	155,125	258	542	Samples isolated in Korea	Samples isolated in China	[110]
	*Duchesnea indica* ^∗^	156,050	45	273	Samples isolated in Korea	Samples isolated in China	[19]
	*Duchesnea chrysantha*	156,29~156,387	48	58	Sample isolated in Korea	Sample isolated in Japan	[21]
	*Pyrus ussuriensis*	159,986~160,157	121	781	Sample isolated in Bonghwa-gun, Korea	Sample isolated in Mt. Hambeak, Korea	[68]
Fagaceae	*Fagus multinervis*	158,349	2	2	Sample isolated in Ulleungdo Island, Korea	Sample isolated in Ulleungdo Island, Korea	[111]
Orobanchaceae	*Rehmannia glutinosa*	153,622~153,680	147	87	Sample isolated in Korea	Sample isolated in China	[73]
Cucurbitaceae	*Cucumis melo*	155,815~156,017	2	0	Cultivar Topmark	Cultivar DHL92	[76]
			1	0	Cultivar MR-1	Cultivar DHL92	
			2	0	Cultivar M4-	Cultivar DHL92	
			1	0	Cultivar M4	Cultivar DHL92	
			55	0	Cultivar M1-1	Cultivar DHL92	
			56	0	Cultivar M1-3	Cultivar DHL92	
			60	0	Cultivar M1-7	Cultivar DHL92	
Amaranthaceae	*Chenopodium quinoa*		69	28	Cultivar 0654 (Peru)	Cultivar PI 614886 (Chile)	[70]
			33	23	Cultivar Cherry Vanilla (Oregon, USA)	Cultivar PI 614886 (Chile)	
			16	37	Cultivar Chucapaca (Bolivia)	Cultivar PI 614886 (Chile)	
			36	23	Cultivar CICA-17 (Peru)	Cultivar PI 614886 (Chile)	
			0	0	Cultivar G-205-95DK (Denmark)	Cultivar PI 614886 (Chile)	
			0	0	Cultivar Ku-2 (Chile)	Cultivar PI 614886 (Chile)	
			36	24	Cultivar Kurmi (Bolivia)	Cultivar PI 614886 (Chile)	
			35	23	Cultivar Ollague (Chile)	Cultivar PI 614886 (Chile)	
			25	13	Cultivar Pasankalla (Peru)	Cultivar PI 614886 (Chile)	
			17	36	Cultivar PI 634921 (Chile)	Cultivar PI 614886 (Chile)	
			37	24	Cultivar Real (Bolivia)	Cultivar PI 614886 (Chile)	
			3	0	Cultivar Regalona (Chile)	Cultivar PI 614886 (Chile)	
			33	23	Cultivar Salcedo INIA (Peru)	Cultivar PI 614886 (Chile)	
	*Dysphania pumilio*	151,960~151,962	25	2	Sample isolated in Anyang, Korea	Sample isolated in Gangseo-gu, Seoul, Korea	[28]
	*Suaeda japonica*	152,109	3	3	Sample isolated in Ganghwa, Korea	Sample isolated in Julpo, Korea	[112]
Eucommiaceae	*Eucommia ulmoides*	163,341~163,586	75	80	Sample isolated in China	Sample isolated in China	[75]
Dioscoreaceae	*Dioscorea polystachya*	153,243~153,292	99	0	FLW genotype	BJXS genotype	[77]
			98	0	TSW genotype	BJXS genotype	
			110	0	YTW genotype	BJXS genotype	
			99	0	XSW genotype	BJXS genotype	
			106	0	NJW genotype	BJXS genotype	
			99	0	MHW genotype	BJXS genotype	
Asteraceae	*Artemisia fukudo*	151,011~151,021	7	11	Sample isolated in Aphaedo, Korea	Sample isolated in Jeungdo, Korea	[113]
Oleaceae	*Abeliophyllum distichum*	155,982~156,019	93	56	Sample isolated in Jincheon-gun, Korea	Cultivar “Okhwang 1-ho”	[90]
			9	11	Cultivar “Okhwang 1-ho”	Cultivar candidate “Dae Ryun”	[91]
			102	63	Sample isolated in Jincheon-gun, Korea	Cultivar candidate “Dae Ryun”	
			9	11	Cultivar candidate “Dae Ryun”	Cultivar candidate “Sang Jae”	[92]
			93	56	Sample isolated in Jincheon-gun, Korea	Cultivar candidate “Sang Jae”	
			1	0	MF407183	Cultivar candidate “Sang Jae”	
Orchidaceae	*Goodyera schlechtendaliana*	153,661~154,438	200	511	Sample isolated in Korea (MK144665)	Sample isolated in Korea with different morphology (MK134679)	[53]
			282	1,060	Sample isolated in Korea (MK144665)	Sample isolated in China (AB893949)	
			827	1,794	Sample isolated in Korea (MK144665)	Sample isolated in China (NC_029364)	
			700	1,066	Sample isolated in Korea (MK144665)	Sample isolated in China (LC085346)	
			163	651	Sample isolated in Korea with different morphology (MK134679)	Sample isolated in China (AB893949)	
			740	1,470	Sample isolated in Korea with different morphology (MK134679)	Sample isolated in China (NC_029364)	
			597	1,065	Sample isolated in Korea with different morphology (MK134679)	Sample isolated in China (LC085346)	
			844	2,045	Sample isolated in China (AB893949)	Sample isolated in China (NC_029364)	
			445	414	Sample isolated in China (AB893949)	Sample isolated in China (LC085346)	
			700	2,133	Sample isolated in China (LC085346)	Sample isolated in China (LC085346)	
	*Gastrodia elata*	35,066~35,304	457	670	Sample isolated in Korea (MN296709)	Sample isolated in China	[56]
			493	650	Sample isolated in Korea (MN296709)	Sample isolated in China	[57]
			324	630	Sample isolated in Korea (MN026874)	Sample isolated in Korea (MN296709)	
Adoxaceae	*Viburnum erosum*	158,587~158,624	16	49	Sample isolated in Korea	Sample isolated in Korea (MN218778)	[64]
Ranunculaceae	*Aconitum coreanum*	157,024~157,040	5	17	Sample isolated in Korea	Sample isolated in Korea (NC_031421)	[89]
			23	62	Sample isolated in Korea	Sample isolated in Korea (KU318669)	
Staphyleaceae	*Euscaphis japonica*	160,467~160,606	424	809	Sample isolated in Korea	Sample isolated in China	(Oh et al., under review)
Thymelaeaceae	*Daphne genkwa*	132,741~132,869	69	650	Sample isolated in Korea	Sample isolated in China	(Oh et al., under review)

∗ indicates that the numbers of SNPs and INDELs were inferred only from LSC, SSC, and IRb regions because one of chloroplast genomes used for comparison is partial genome.

**Table 4:** Number of SSRs and extendedSSRs along with SSR types and its origin.

SSR type	180404IB4	15-11	Kyoto	Tsu0	Ler0	Col0
MonoSSR	67	69	68	70	67	69
DiSSR	17	17	18	16	18	18
TriSSR	6	6	6	6	6	6
TetraSSR	8	8	9	8	9	9
PentaSSR	2	3	2	1	3	2
HexaSSR	1	1	1	1	0	1
HeptaSSR	27	25	25	25	26	25
OctaSSR	8	8	9	10	8	7
NonaSSR	6	6	6	6	6	6
DecaSSR	1	1	1	1	1	1
Total	143	144	145	144	144	144

## Data Availability

Chloroplast genome sequence of Korean *A. thaliana* can be accessed via accession number MK353213 in NCBI GenBank. In addition, three more chloroplast genomes of *A. thaliana*, Kyoto, Tsu0, and 11-15, based on SRA datasets are accessible through MK380720, MK380721, and MK380719, respectively.

## Supplementary Materials

Supplementary Materials List of SSRs identified from *A. thaliana* 180404IB4 chloroplast genome.
