# Supplementary material for: The Complete Chloroplast Genome of Arabidopsis thaliana Isolated in Korea (Brassicaceae): An Investigation of Intraspecific Variations of the Chloroplast Genome of Korean A. thaliana
Source: Int J Genomics. 2020 Sep 5;2020:3236461. doi: 10.1155/2020/3236461 (PMC7492873; doi:10.1155/2020/3236461)
Supplement: Supplementary Materials — List of SSRs identified from A. thaliana 180404IB4 chloroplast genome. [file 3236461.f1.docx]

**Supplementary Table 1 List of SSRs identified from *A. thaliana* 180404IB4 chloroplast genome**

| **No** | **Name** | **SSR type** | **Type** | **Cooridnation** | | **Unit sequence** | **Repeat number** | **Position** | **Genes** |
| --- | --- | --- | --- | --- | --- | --- | --- | --- | --- |
| 1 | cM0000001 | SSR | MonoSSR | 112 | 126 | A | 15 | Intergenic |  |
| 2 | c70000001 | ExtendedSSR | HeptaSSR | 137 | 150 | AATAAAA | 2 | Intergenic |  |
| 3 | cT0000001 | SSR | TriSSR | 213 | 224 | TTA | 4 | Intergenic |  |
| 4 | c100000001 | ExtendedSSR | DecaSSR | 1691 | 1710 | TGAATACGAT | 2 | Intergenic |  |
| 5 | cM0000002 | SSR | MonoSSR | 1781 | 1790 | A | 10 | Intronic | trnK |
| 6 | c90000001 | ExtendedSSR | NonaSSR | 3445 | 3462 | TCTATTTAA | 2 | Exonic | matK,trnK |
| 7 | cD0000001 | SSR | DiSSR | 3825 | 3834 | AT | 5 | Intronic | trnK |
| 8 | cM0000003 | SSR | MonoSSR | 4114 | 4125 | T | 12 | Intronic | trnK |
| 9 | cM0000004 | SSR | MonoSSR | 4353 | 4362 | A | 10 | Intergenic |  |
| 10 | cTe0000001 | SSR | TetraSSR | 4526 | 4537 | AAAT | 3 | Intergenic |  |
| 11 | c70000004 | ExtendedSSR | HeptaSSR | 4657 | 4670 | ATTTTTT | 2 | Intergenic |  |
| 12 | cM0000005 | SSR | MonoSSR | 4729 | 4739 | T | 11 | Intergenic |  |
| 13 | cTe0000002 | SSR | TetraSSR | 6371 | 6382 | ATAA | 3 | Intergenic |  |
| 14 | cM0000006 | SSR | MonoSSR | 7905 | 7917 | A | 13 | Intergenic |  |
| 15 | cD0000003 | SSR | DiSSR | 7995 | 8008 | TA | 7 | Intergenic |  |
| 16 | cD0000004 | SSR | DiSSR | 8054 | 8067 | TA | 7 | Intergenic |  |
| 17 | c80000004 | ExtendedSSR | OctaSSR | 8073 | 8088 | TTTGATTA | 2 | Intergenic |  |
| 18 | cD0000005 | SSR | DiSSR | 8094 | 8103 | AT | 5 | Intergenic |  |
| 19 | cD0000006 | SSR | DiSSR | 8122 | 8131 | AT | 5 | Intergenic |  |
| 20 | c90000002 | ExtendedSSR | NonaSSR | 8145 | 8162 | TATATAAAT | 2 | Intergenic |  |
| 21 | cD0000007 | SSR | DiSSR | 8170 | 8179 | TA | 5 | Intergenic |  |
| 22 | cD0000008 | SSR | DiSSR | 8180 | 8189 | AT | 5 | Intergenic |  |
| 23 | cD0000009 | SSR | DiSSR | 8205 | 8214 | AT | 5 | Intergenic |  |
| 24 | c70000008 | ExtendedSSR | HeptaSSR | 9579 | 9592 | AAAAGCG | 2 | Exonic.Intronic | trnR-TCT |
| 25 | cM0000007 | SSR | MonoSSR | 12843 | 12852 | T | 10 | Intergenic |  |
| 26 | cT0000002 | SSR | TriSSR | 12978 | 12989 | AAT | 4 | Intergenic |  |
| 27 | c70000011 | ExtendedSSR | HeptaSSR | 13920 | 13933 | AATATCA | 2 | Intergenic |  |
| 28 | cM0000008 | SSR | MonoSSR | 14008 | 14017 | A | 10 | Intergenic |  |
| 29 | c80000006 | ExtendedSSR | OctaSSR | 14831 | 14846 | TTTACTTA | 2 | Intergenic |  |
| 30 | cM0000009 | SSR | MonoSSR | 14979 | 14988 | A | 10 | Intergenic |  |
| 31 | cM0000010 | SSR | MonoSSR | 17926 | 17938 | T | 13 | Exonic | rpoC2 |
| 32 | cD0000010 | SSR | DiSSR | 19298 | 19307 | TA | 5 | Exonic | rpoC2 |
| 33 | cM0000011 | SSR | MonoSSR | 25684 | 25693 | T | 10 | Exonic | rpoB |
| 34 | cM0000012 | SSR | MonoSSR | 28268 | 28277 | T | 10 | Intergenic |  |
| 35 | c80000007 | ExtendedSSR | OctaSSR | 28307 | 28322 | TTTTCTAT | 2 | Intergenic |  |
| 36 | cTe0000003 | SSR | TetraSSR | 28414 | 28425 | CAAA | 3 | Intergenic |  |
| 37 | cM0000013 | SSR | MonoSSR | 28673 | 28685 | T | 13 | Intergenic |  |
| 38 | cT0000003 | SSR | TriSSR | 28823 | 28834 | TTA | 4 | Intergenic |  |
| 39 | cM0000014 | SSR | MonoSSR | 28835 | 28844 | T | 10 | Intergenic |  |
| 40 | cM0000015 | SSR | MonoSSR | 29060 | 29069 | A | 10 | Intergenic |  |
| 41 | c70000012 | ExtendedSSR | HeptaSSR | 30228 | 30241 | CTATACC | 2 | Intergenic |  |
| 42 | cM0000016 | SSR | MonoSSR | 30287 | 30299 | A | 13 | Intergenic |  |
| 43 | c70000013 | ExtendedSSR | HeptaSSR | 30636 | 30649 | TTTTTTC | 2 | Intergenic |  |
| 44 | cM0000017 | SSR | MonoSSR | 30667 | 30676 | T | 10 | Intergenic |  |
| 45 | cM0000018 | SSR | MonoSSR | 30831 | 30842 | A | 12 | Intergenic |  |
| 46 | cM0000019 | SSR | MonoSSR | 30866 | 30875 | T | 10 | Intergenic |  |
| 47 | cM0000020 | SSR | MonoSSR | 31017 | 31029 | T | 13 | Intergenic |  |
| 48 | cD0000011 | SSR | DiSSR | 31309 | 31324 | AT | 8 | Intergenic |  |
| 49 | c80000009 | ExtendedSSR | OctaSSR | 31665 | 31680 | TTTTTCGA | 2 | Intergenic |  |
| 50 | cM0000021 | SSR | MonoSSR | 32370 | 32379 | A | 10 | Intergenic |  |
| 51 | c70000014 | ExtendedSSR | HeptaSSR | 32381 | 32394 | TTCTTTA | 2 | Intergenic |  |
| 52 | cP0000065 | SSR | PentaSSR | 36258 | 36272 | ATTAA | 3 | Intergenic |  |
| 53 | cD0000012 | SSR | DiSSR | 36331 | 36340 | AT | 5 | Intergenic |  |
| 54 | cD0000013 | SSR | DiSSR | 36346 | 36361 | AT | 8 | Intergenic |  |
| 55 | cM0000022 | SSR | MonoSSR | 36693 | 36702 | A | 10 | Intergenic |  |
| 56 | c80000011 | ExtendedSSR | OctaSSR | 41972 | 41987 | CATCTATT | 2 | Intergenic |  |
| 57 | cM0000023 | SSR | MonoSSR | 42555 | 42565 | A | 11 | Intergenic |  |
| 58 | c70000015 | ExtendedSSR | HeptaSSR | 43044 | 43057 | TATTAGA | 2 | Intronic | ycf3 |
| 59 | cT0000004 | SSR | TriSSR | 44132 | 44143 | GAA | 4 | Intronic | ycf3 |
| 60 | cM0000024 | SSR | MonoSSR | 45196 | 45206 | T | 11 | Intergenic |  |
| 61 | cTe0000004 | SSR | TetraSSR | 46444 | 46455 | TAAA | 3 | Intergenic |  |
| 62 | cD0000014 | SSR | DiSSR | 46471 | 46480 | TA | 5 | Intergenic |  |
| 63 | cT0000005 | SSR | TriSSR | 46580 | 46594 | AAT | 5 | Intergenic |  |
| 64 | cM0000025 | SSR | MonoSSR | 46615 | 46628 | A | 14 | Intergenic |  |
| 65 | cM0000026 | SSR | MonoSSR | 46672 | 46681 | A | 10 | Intergenic |  |
| 66 | c70000017 | ExtendedSSR | HeptaSSR | 48077 | 48090 | TGATACT | 2 | Intergenic |  |
| 67 | c70000018 | ExtendedSSR | HeptaSSR | 48389 | 48402 | ATACATA | 2 | Intergenic |  |
| 68 | cM0000027 | SSR | MonoSSR | 49959 | 49971 | T | 13 | Intergenic |  |
| 69 | cM0000028 | SSR | MonoSSR | 50846 | 50856 | A | 11 | Intergenic |  |
| 70 | c70000019 | ExtendedSSR | HeptaSSR | 50954 | 50967 | TAAATTA | 2 | Intergenic |  |
| 71 | cM0000029 | SSR | MonoSSR | 54101 | 54110 | T | 10 | Exonic | atpB |
| 72 | c70000020 | ExtendedSSR | HeptaSSR | 56636 | 56649 | ATTCTTA | 2 | Intergenic |  |
| 73 | cM0000030 | SSR | MonoSSR | 56839 | 56848 | T | 10 | Intergenic |  |
| 74 | cM0000031 | SSR | MonoSSR | 57372 | 57384 | A | 13 | Exonic | accD |
| 75 | c70000021 | ExtendedSSR | HeptaSSR | 58839 | 58852 | TTCTAAT | 2 | Intergenic |  |
| 76 | c70000022 | ExtendedSSR | HeptaSSR | 58901 | 58914 | ACTATTA | 2 | Intergenic |  |
| 77 | cH0000074 | SSR | HexaSSR | 59041 | 59058 | TTCTAT | 3 | Intergenic |  |
| 78 | c90000004 | ExtendedSSR | NonaSSR | 59138 | 59155 | ATATAATAG | 2 | Intergenic |  |
| 79 | cM0000032 | SSR | MonoSSR | 59387 | 59397 | T | 11 | Intergenic |  |
| 80 | cM0000033 | SSR | MonoSSR | 60382 | 60391 | T | 10 | Intergenic |  |
| 81 | cD0000015 | SSR | DiSSR | 63122 | 63137 | AT | 8 | Intergenic |  |
| 82 | cM0000034 | SSR | MonoSSR | 63164 | 63176 | T | 13 | Intergenic |  |
| 83 | cTe0000005 | SSR | TetraSSR | 63194 | 63205 | TAAA | 3 | Intergenic |  |
| 84 | c70000023 | ExtendedSSR | HeptaSSR | 63248 | 63261 | AAATTTT | 2 | Intergenic |  |
| 85 | cP0000119 | SSR | PentaSSR | 64858 | 64872 | TTGGA | 3 | Intergenic |  |
| 86 | c70000026 | ExtendedSSR | HeptaSSR | 65041 | 65054 | AAAACCT | 2 | Intergenic |  |
| 87 | cTe0000006 | SSR | TetraSSR | 65126 | 65137 | AAAT | 3 | Intergenic |  |
| 88 | cM0000035 | SSR | MonoSSR | 66308 | 66320 | T | 13 | Intergenic |  |
| 89 | cM0000036 | SSR | MonoSSR | 66700 | 66715 | T | 16 | Intergenic |  |
| 90 | c70000027 | ExtendedSSR | HeptaSSR | 66904 | 66917 | AAAGAAG | 2 | Intergenic |  |
| 91 | cM0000037 | SSR | MonoSSR | 67164 | 67176 | G | 13 | Intergenic |  |
| 92 | c70000028 | ExtendedSSR | HeptaSSR | 67304 | 67317 | AAATCAT | 2 | Intergenic |  |
| 93 | cM0000038 | SSR | MonoSSR | 68340 | 68350 | A | 11 | Intergenic |  |
| 94 | cM0000039 | SSR | MonoSSR | 70189 | 70201 | A | 13 | Intronic | rps12,clpP |
| 95 | cM0000040 | SSR | MonoSSR | 70404 | 70416 | T | 13 | Intronic | rps12,clpP |
| 96 | cM0000041 | SSR | MonoSSR | 71178 | 71188 | T | 11 | Intronic | rps12,clpP |
| 97 | c70000031 | ExtendedSSR | HeptaSSR | 72919 | 72932 | GGCGTGG | 2 | Exonic | psbB,rps12 |
| 98 | c90000005 | ExtendedSSR | NonaSSR | 74955 | 74972 | AAATAGATC | 2 | Intronic | rps12 |
| 99 | cM0000042 | SSR | MonoSSR | 76735 | 76746 | A | 12 | Intronic | rps12 |
| 100 | cTe0000007 | SSR | TetraSSR | 77782 | 77797 | TTAA | 4 | Intronic | rps12 |
| 101 | cM0000043 | SSR | MonoSSR | 78095 | 78108 | T | 14 | Exonic | rpoA,rps12 |
| 102 | c70000032 | ExtendedSSR | HeptaSSR | 78572 | 78585 | GCGCGTT | 2 | Exonic | rpoA,rps12 |
| 103 | cM0000044 | SSR | MonoSSR | 82454 | 82464 | T | 11 | Intronic | rps12,rpl16 |
| 104 | cM0000045 | SSR | MonoSSR | 82601 | 82610 | T | 10 | Intronic | rps12,rpl16 |
| 105 | cD0000016 | SSR | DiSSR | 94513 | 94522 | TA | 5 | Intronic | rps12 |
| 106 | c70000034 | ExtendedSSR | HeptaSSR | 94817 | 94830 | AGATTAT | 2 | Intronic | rps12 |
| 107 | cM0000046 | SSR | MonoSSR | 99358 | 99374 | A | 17 | Intronic | rps12 |
| 108 | cM0000047 | SSR | MonoSSR | 99394 | 99404 | T | 11 | Intronic | rps12 |
| 109 | cM0000048 | SSR | MonoSSR | 100162 | 100172 | T | 11 | Intronic | rps12 |
| 110 | c80000012 | ExtendedSSR | OctaSSR | 104356 | 104371 | TTTTGAGA | 2 | Intronic | rps12,trnA |
| 111 | c70000036 | ExtendedSSR | HeptaSSR | 108113 | 108126 | AAAAATG | 2 | Intronic | rps12 |
| 112 | c90000007 | ExtendedSSR | NonaSSR | 108521 | 108538 | TATATCTAT | 2 | Intronic | rps12 |
| 113 | cM0000049 | SSR | MonoSSR | 110408 | 110417 | A | 10 | Exonic | ycf1,ndhF,rps12 |
| 114 | cTe0000008 | SSR | TetraSSR | 112453 | 112464 | ATAG | 3 | Exonic | ndhF,rps12 |
| 115 | cD0000017 | SSR | DiSSR | 112691 | 112700 | TA | 5 | Intronic | rps12 |
| 116 | cM0000050 | SSR | MonoSSR | 113281 | 113290 | A | 10 | Intronic | rps12 |
| 117 | c80000013 | ExtendedSSR | OctaSSR | 114423 | 114438 | TATTAATT | 2 | Intronic | rps12 |
| 118 | c70000037 | ExtendedSSR | HeptaSSR | 115601 | 115614 | CAAAGAC | 2 | Intronic | rps12 |
| 119 | cM0000051 | SSR | MonoSSR | 115640 | 115649 | T | 10 | Intronic | rps12 |
| 120 | cM0000052 | SSR | MonoSSR | 119057 | 119067 | T | 11 | Intronic | rps12 |
| 121 | cT0000006 | SSR | TriSSR | 119149 | 119163 | TAT | 5 | Intronic | rps12 |
| 122 | c70000039 | ExtendedSSR | HeptaSSR | 119497 | 119510 | TTTCCAA | 2 | Exonic | ndhI,rps12 |
| 123 | cM0000053 | SSR | MonoSSR | 120668 | 120678 | A | 11 | Intronic | rps12,ndhA |
| 124 | cM0000054 | SSR | MonoSSR | 120779 | 120788 | A | 10 | Intronic | rps12,ndhA |
| 125 | c70000040 | ExtendedSSR | HeptaSSR | 121144 | 121157 | CCGGTTA | 2 | Intronic | rps12,ndhA |
| 126 | cM0000055 | SSR | MonoSSR | 124121 | 124132 | T | 12 | Exonic | ycf1,rps12 |
| 127 | cM0000056 | SSR | MonoSSR | 124214 | 124224 | T | 11 | Exonic | ycf1,rps12 |
| 128 | cM0000057 | SSR | MonoSSR | 124287 | 124296 | T | 10 | Exonic | ycf1,rps12 |
| 129 | cM0000058 | SSR | MonoSSR | 125808 | 125817 | T | 10 | Exonic | ycf1,rps12 |
| 130 | cM0000059 | SSR | MonoSSR | 125861 | 125873 | T | 13 | Exonic | ycf1,rps12 |
| 131 | cM0000060 | SSR | MonoSSR | 126010 | 126020 | A | 11 | Exonic | ycf1,rps12 |
| 132 | cM0000061 | SSR | MonoSSR | 127017 | 127026 | T | 10 | Exonic | ycf1,rps12 |
| 133 | cM0000062 | SSR | MonoSSR | 127047 | 127057 | T | 11 | Exonic | ycf1,rps12 |
| 134 | cM0000063 | SSR | MonoSSR | 127077 | 127088 | A | 12 | Exonic | ycf1,rps12 |
| 135 | cM0000064 | SSR | MonoSSR | 128218 | 128227 | T | 10 | Exonic | ycf1,rps12 |
| 136 | c90000008 | ExtendedSSR | NonaSSR | 130097 | 130114 | ATAGATATA | 2 | Intronic | rps12 |
| 137 | c70000041 | ExtendedSSR | HeptaSSR | 130505 | 130518 | TTTTCAT | 2 | Intronic | rps12 |
| 138 | c80000014 | ExtendedSSR | OctaSSR | 134264 | 134279 | TCTCAAAA | 2 | Intronic | rps12,trnA |
| 139 | cM0000065 | SSR | MonoSSR | 138463 | 138473 | A | 11 | Intronic | rps12 |
| 140 | cM0000066 | SSR | MonoSSR | 139231 | 139241 | A | 11 | Intronic | rps12 |
| 141 | cM0000067 | SSR | MonoSSR | 139261 | 139277 | T | 17 | Intronic | rps12 |
| 142 | c70000043 | ExtendedSSR | HeptaSSR | 143804 | 143817 | TATAATC | 2 | Intergenic |  |
| 143 | cD0000018 | SSR | DiSSR | 144112 | 144121 | AT | 5 | Intergenic |  |
